# Introduction of High-Fidelity Simulation (HFS) for Teaching Undergraduate Medical Students About Electro convulsive Therapy (ECT) and Its Impact on Their Knowledge and Attitudes Towards ECT

**DOI:** 10.1192/bjo.2024.238

**Published:** 2024-08-01

**Authors:** Vishi Sachdeva, Supriya Dastidar, Meena Murugan, Thomas Rourke, Jasleen Deol

**Affiliations:** Birmingham and Solihull Mental Health Foundation Trust, Birmingham, United Kingdom

## Abstract

**Aims:**

Learning about and appreciating the use of Electroconvulsive therapy remains an integral part of the undergraduate psychiatry curriculum. The existing literature indicates that medical students frequently have unfavourable views regarding ECT and its adverse effects.

Therefore, this study aimed to introduce a new teaching tool that supplements traditional didactic ECT teaching with simulation-based procedural demonstration thus providing a real-life experience of an ECT room and subsequently evaluate the learning gains conferred by such a curriculum.

**Methods:**

The demonstration was carried out by Clinical teaching Fellow with the help of a high-fidelity manikin and a live actor who played the role of the patient, in the ECT suite in Birmingham.

Participants of the study were fourth year medical students who completed a self-administered questionnaire before and after the simulation session. This survey was designed to explore changes in knowledge, attitudes, and perceptions of the students towards ECT and its side effects.

**Results:**

Within a cohort of 88 students, 52 students successfully completed the pre-session questionnaire, and 43 students completed the post-session questionnaire. Students reported a global improvement in knowledge regarding ECT, when comparing results from both questionnaires. Prior to the simulation, many students used negative terms to describe ECT such as ‘torture’, ‘barbaric’ and ‘uncontrolled’, suggesting outdated stigmas around ECT. However, after the simulation, many students expressed a positive change in opinion, describing ECT as ‘controlled’, ‘beneficial’ and effective’.

Additionally, students reported improved knowledge about the side effects of ECT, especially regarding pain, memory loss and brain injury. Many students reported that their initial apprehension had been addressed over the course of the ECT simulation. Many noted ECT was more effective and beneficial than originally thought and the process was less extreme and invasive than they believed.

**Conclusion:**

The results of the study reflect that the use of simulated ECT within medical students can help disperse some of the stigma and myths regarding this treatment. Simulation can humanise the process and shift attitudes around ECT by allowing students to become fully immersed into an almost real-life scenario. It can also address knowledge gaps around ECT indications, process, risks, side effects and benefits. This will in turn help educate future clinicians have a better understanding about ECT in the treatment of severe mental illness, thus optimising the utilisation of this effective treatment. Furthermore, such technique can be a useful tool for demonstrating ECT to potentially wider group of students, trainees and other health practitioners.

